# College students' cyberloafing and the sense of meaning of life: The mediating role of state anxiety and the moderating role of psychological flexibility

**DOI:** 10.3389/fpubh.2022.905699

**Published:** 2022-07-26

**Authors:** Qing Li, Bingnan Xia, Huijia Zhang, Wei Wang, Xiaochen Wang

**Affiliations:** ^1^School of Marxism, Communication University of Zhejiang, Hangzhou, China; ^2^School of Business Administration, Zhejiang Gongshang University, Hangzhou, China; ^3^Office of Academic Research, Zhejiang Gongshang University Hangzhou College of Commerce, Hangzhou, China; ^4^Hangzhou Zhongxing Hospital, Hangzhou, China

**Keywords:** college students, cyberloafing, the sense of meaning of life, psychological flexibility, state anxiety

## Abstract

**Background:**

With the gradual penetration of network media into various fields of people's life, the relationship between network behavior and the sense of meaning of life is bound to be closer and closer. The purpose of this study is to explore the mediating role of state anxiety between cyber loafing and the sense of meaning of life, and the moderating role of psychological flexibility in this mediating relationship.

**Methodology:**

With 964 undergraduates recruited as subjects three-wave-time-lagged quantitative research design was conducted in China. All participants were required to complete a self-reported electronic questionnaire. Then, the mediating mechanism and moderating effect were explored with utilization of SPSS25.0.

**Results:**

The results showed that cyberloafing had significant negative correlation with the sense of meaning of life. Our analysis testing the mediating effect showed that state anxiety partially mediated the relationship between cyberloafing and the sense of meaning of life (indirect effect = −0.05, *p* < 0.01,), while the mediating effect was 31.25% of the total effect. Our analysis testing the moderating effect showed that psychological flexibility significantly moderated the relationship between cyberloafing and state anxiety (interaction effect = −0.26, *p* < 0.01). And our analysis testing the moderated mediating effect showed that psychological flexibility played a moderating role in the mediating effect of state anxiety.

**Conclusion:**

Based on the findings of this study, college students' cyberloafing negatively affects their sense of meaning of life. Therefore, appropriate measures should be taken to supervise and restrict college students' Internet use and provide them with corresponding guidance; certain psychological adjustment measures should also be taken when necessary to help college students with low psychological flexibility in reducing their state anxiety and improving their sense of meaning of life.

## Introduction

With the leaping development of Internet technology, the widespread use of computers, mobile phones and other devices in the e-learning environment has not only created great convenience for learning, but also prompted college students to enter the era of pan-entertainment. The use of electronic media by college students not for the purpose of learning, in or out of class when completing learning tasks (1, 2) is referred to as cyberloafing in learning. Researchers believe that cyberloafing is a problematic behavior of network use (3), and it has become a prevailing topic in the field of psychological research exploring the impact of this commonly seen network behavior among college students.

Cyberloafing was originally used to describe the phenomenon that employees use the network tools provided by the organization to browse non-work-related websites, sending and receiving personal e-mails, which has an impact on job performance during working hours. Recent research studies on cyberloafing mainly focus on the field of organizational behaviors in firms. However, some studies have revealed that cyberloafing among college students, which has deviated them from learning, is even more serious than that among employees (1). However, empirical studies on college students' cyberloafing mainly focus on the impact of college students' cyberloafing on their academic performance and mental health. For example, college students' cyberloafing could lead to decreased attention toward their academic work and poor performance in learning (4, 5). Also, cyberloafing is negatively correlated with subjective wellbeing (6), which can lead to depression (7). Such studies mainly focus on the outcome variables of cyberloafing, leaving the internal mechanism of this negative impact caused by cyberloafing insufficiently explored. Therefore, this study would provide certain theoretical value and practical significance through expanding the research on the impact of college students' cyberloafing and revealing related internal mechanisms.

### The relationship between cyberloafing and the sense of meaning of life

Current research evidence still show some inconsistencies on the impact caused by cyberloafing. Among these researches, some believe that cyberloafing has a negative impact on both organizations and employees. For example, it is found that cyberloafing will lead to loss of organizational productivity (8) and psychological stress of employees (9); it also triggers negative emotions (10) and reduces job satisfaction among employees (11). Nonetheless, some researchers believe that cyberloafing can promote the recovery of work, temporarily reduce pressure and mental tension brought by work; through providing a feeling of relaxation to employees, cyberloafing can improve employees' devotion to follow-up work; this is because cyberloafing can further complement important emotional resources for employees and indirectly promotes their efficiency in achieving work tasks (12). Research on college students' cyberloafing showed that using the Internet in class was related to lower emotional wellbeing, which showed more depression symptoms and higher social anxiety (13). However, some studies have found that students would experience more positive emotions and less negative emotions after participating in cyberloafing (14). Therefore, it is necessary to explore the impact of cyberloafing on individual psychology and behavior.

The social displacement hypothesis holds that the use of network media encroaches on people's time for social interaction and other activities in their daily life and makes it easier for them to fall into depression and suffer alienation, which would eventually reduce individual sense of happiness (15). For college students, cyberloafing takes up a large portion of their time which should have been devoted to learning and consumes certain amount of scarce attention resources (16). The sense of meaning of life is understood as having a strong sense of life purpose, pursuing valuable personal goals, or having a clear value system that can guide one's actions (17). Internet addiction negatively predicted the sense of meaning of life (18). With the gradual penetration of network media into all fields of people's life, the relationship between network behavior and the sense of meaning of life is bound to be closer and closer. However, at present, few studies pay attention to the relationship between cyberloafing and the sense of meaning of life. Therefore, we presume that cyberloafing may create anxiety and trigger confusion as regard to the value and goal of life and learning, and put forward the following hypothesis.

H1: Cyberloafing significantly and negatively predict the sense of meaning of life.

### The mediating role of state anxiety

State anxiety refers to the state of immediate anxiety in a specific scene at present or in a specific period of time (19). When an individual encounters external or internal stimulus (such as thought, psychological needs, etc.), the existence of threat can be felt by his or her body, and the emotion of state anxiety will arise. Researchers have pointed out that cyberloafing will increase employees' work anxiety, thus depleting their emotional resources and weakening their sense of meaning in work (20). In addition, excessive use of the Internet will have a negative impact on the physical and mental health of individuals, such as the increase of anxiety and depression (13). Previous studies have shown that addiction to social media would have a negative impact on individuals. For example, excessive use of Wechat (a social media platform in China which is equivalent to twitter) will also affect teenagers' physical and mental health, as well as their academic performance (21). According to the stress transactional model of Lazarus and Folkman (22), when people think the events they experience are harmful or threatening, exposure to these pressures will lead to negative physical (such as elevated blood pressure), psychological (anger and anxiety) or behavioral (leaving the status quo) results. According to the research by Becker et al. (13) students who used the Internet in class exhibited more depressive symptoms, higher stress and anxiety. Thus, it can be inferred that college students' cyberloafing during learning can be regarded as an investment of limited learning resources (such as time and energy) in non-learning fields. This irrational allocation of learning resources can be problematic, which would lead to increased psychological pressure and anxiety. This transient experience of tension in learning can be very easily changed with variation of the surrounding environment. This is a typical manifestation of psychological distress leading to resource loss (23). Therefore, this study puts forward the following hypothesis.

H2: Cyberloafing is positively correlated with state anxiety.

With occurrence of anxiety in social communication, college students' perceived subjective wellbeing will also decrease (24). The higher one's state anxiety level is, the lower one's sense of wellbeing is, and the sense of wellbeing is positively correlated with the sense of meaning of life (25). In addition, anxiety is a kind of negative emotion. Negative emotional experience increases the risk of anxiety and depression (26), which would diminish their sentiment in the sense of meaning of life (27, 28). Zhang et al. (29) found that there was a significant negative correlation between negative indicators of mental health (such as trait anxiety) and the sense of meaning of life. Individuals with trait anxiety are easily attracted by negative emotional information in the environment, which leads to habitual indulgence in anxiety, resulting in state anxiety, and then in reduced the sense of meaning of life (30). Thus, we propose that cyberloafing will intensify college students' state anxiety and reduce their sense of meaning of life. Therefore, the following assumptions are put forward.

H3: State anxiety has a negative impact on the sense of meaning of life.
H4: State anxiety mediates the relationship between cyberloafing and the sense of meaning of life.

### The moderating role of psychological flexibility

According to the organism-environment interaction model, individuals develop differently (more or less sensitive) to similar environments depending on certain intrinsic personal attributes, and thus show different adaptive outcomes (31). Yet, there are no empirical studies examining the moderating role of individual intrinsic attributes in the direct or indirect relationship between online loafing and sense of meaning in life. Psychological flexibility means that individuals consciously contact the present situation in a flexible and independent way, and act according to their own values in light of the conditions provided by the environment (32). Individuals with psychological flexibility pay attention to their current life experience and feelings with a receptive attitude, by observing those conceptualized self- experiences from the perspective of a third party, and actively dealing with negative emotional events. As a protective factor (33, 34), psychological flexibility may affect the link between state anxiety and mental health through a moderating effect and can help resist the adverse effects caused by trauma (35). It is considered as an adaptive coping strategy and plays an important role in recovering from adversity and stress (36). Therefore, cyberloafing, individuals with high level of psychological flexibility can pay attention to the current experience and feelings with a receptive attitude, which lead to improved physical and mental functions and reduced level of state anxiety; in comparison, individuals with low psychological flexibility have a more rigid thinking mode, which would lead to a tendency to fall into and be trapped with negative emotions and cognition, thus intensifying their state anxiety. Based on the analysis above, this study puts forward the following assumptions.

H5: Psychological flexibility negatively moderates the relationship between cyberloafing and state anxiety. The stronger an individual's psychological flexibility, the weaker the positive relationship between cyberloafing and state anxiety. And the weaker an individual's psychological flexibility, the stronger the positive relationship between cyberloafing and state anxiety.

**Figure 1:**
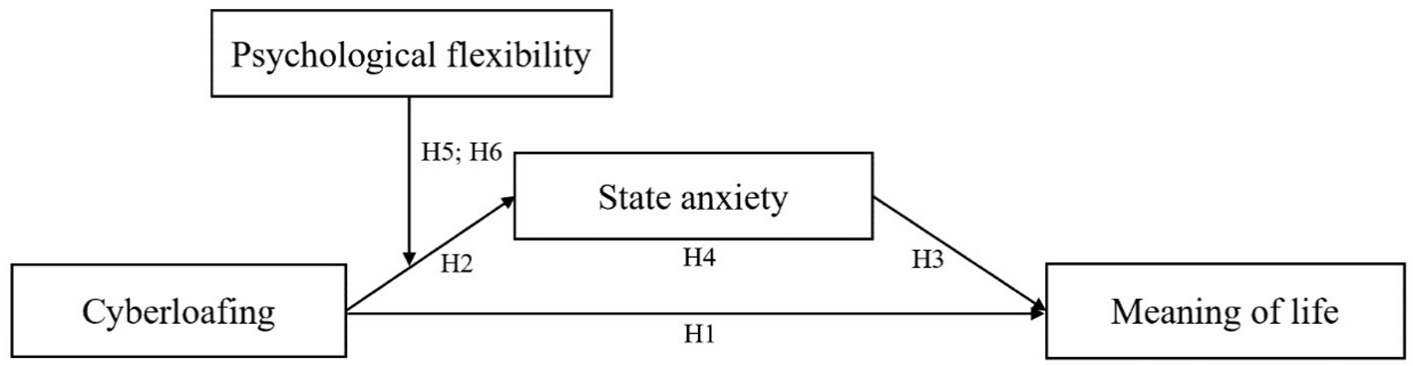
Theoretical model.

The relationships demonstrated by H4 and H5 further reveals the moderated mediation model, that is, the mediating effect of state anxiety between cyberloafing and the sense of meaning of life is strengthened under the condition of low psychological flexibility. Based on the above, this study constructs a moderated mediation model with an overall theoretical framework shown in Figure 1.

H6: Psychological flexibility moderates the mediating effect of state anxiety between cyberloafing and sense of life meaning, that is, the stronger the psychological flexibility of college students, the weaker the mediating effect of state anxiety; while the weaker the psychological flexibility of college students, the stronger the mediating effect of state anxiety.

## Methods

### Participants

The study hosted a questionnaire on an online survey platform (Survey Star, www.wjx.com). Completion of the questionnaire was voluntary and anonymity was assured. Since common method bias may inflate the correlations among variables and reduce the accuracy of our conclusions, we created a temporal separation by introducing a time lag between the measurement (37). Our data was collected at three phases, separated by about 2 weeks. In Time-1 (End of October 2021), participants were requested respond to some demographic questions (including gender, age and the experience of using the network) and to evaluate the level of cyberloafing and their own level of psychological flexibility. One thousand one hundred and thirty seven questionnaires were collected, and 1,109 valid questionnaires were obtained after excluding invalid ones. In Time-2 (Mid November 2021), the survey was conducted following the same procedures, with participants being requested to assess their state anxiety. In this phase, 1,031 valid samples were collected. In Time-3 (Early December 2021), participants were requested to evaluate their sense of meaning of life. Finally, a total of 964 valid questionnaires were obtained. The overall data return rate was 84.8%.

Among the valid participants, 39.32% were male, 60.68% were female, the average age was 18.67 years (SD = 1.36). As for the experience of using network, 12.1% of participating students had an experience of network using of <5 years; 53.2% of them had 5-to-10-year experience, and 34.6% of them have used network for more than 10 years.

### Measures

#### Cyberloafing

Cyberloafing was measured by the original cyberloafing scale, which was designed by Van Doorn (38). The questionnaire consisted of two dimensions and only the activity dimension was used in our study. The activity dimension was referred to as the specific activities that individuals can participate through the Internet, which could be divided into four types (social activities, information activities, leisure activities and virtual emotional activities). The activity dimension subscale contained 12 items, 3 for each activity type; for example: “I engage in cyberloafing in order to maintain social network” (social activity); “I engage in cyberloafing in order to search for information” (informational activity); “I engage in cyberloafing in order to play an online game” (leisure activity); “I engage in cyberloafing in order to shop online” (virtual emotional activity). The scale was scored from 1 (strongly disagree) to 5 (strongly agree). The Cronbach's α for this measure was 0.89.

#### State anxiety

State anxiety was measured by the subscale of the State-Trait Anxiety Inventory (STAI) (39), which was revised by Li and Qian (40). The questionnaire consisted of 20 items, including 20 sentences (10 sentences describing negative emotions and 10 sentences describing positive emotions). An example item was “I feel afraid.” The scale was scored from 1 (strongly disagree) to 5 (strongly agree). The higher the score, the more severe the state anxiety; among the questions, 10 of them were reversely scored. The Cronbach's α for this measure was 0.92.

#### The sense of meaning of life

The sense of meaning of life was measured by the Meaning of Life Questionnaire (MLQ), which consisted of two aspects, the presence of meaning and the search for meaning. The scale measured the individual's experience of meaning in life and the motivation to search for meaning in life, respectively. Each subscale contained 5 items, such as “I understand my life's meaning” and “I am looking for something that makes my life feel meaningful.” The scale was scored from 1 (strongly disagree) to 5 (strongly agree) and higher scores indicate higher sense of meaning of life (41). The Cronbach's α for the overall scale was 0.91.

#### Psychological flexibility

Psychological flexibility was measured by the Acceptance and Action Questionnaire-2nd Edition (AAQ-II) (42), which was revised by Cao et al. (43) and had been extensively tested for reliability in China. The questionnaire consisted of 7 items. An example item was “It seems like most people are handling their lives better than I am.” The scale was scored from 1 (strongly disagree) to 5 (strongly agree), with higher scores after reverse scoring suggesting greater psychological flexibility. The Cronbach's α for the scale was 0.84.

#### Control variables

In this study, we controlled several demographic characteristics including gender, age and the experience of using the network. Gender was coded as a dummy variable (1 = male, 2 = female). Age was measured by the number of years. The experience of using the network was divided into 3 levels (1 = <5 years, 2 = 5 to 10 years, 3 = more than 10 years).

### Data analysis

SPSS 25.0 and Amos 26.0 were used for data analysis and testing. Also, the Bootstrap method *via* SPSS macro program PROCESS V3.3 was applied. Firstly, in order to test the validity and calculate the Cronbach alpha coefficient to estimate internal consistency, confirmatory factor analysis was conducted by AMOS. Secondly, descriptive statistics and Pearson correlations were calculated among variables. Thirdly, PROCESS macro for SPSS (Model 4) was applied to examine the mediating effect of state anxiety, and PROCESS macro (Model 7) was applied to examine the moderating effect of psychological flexibility on the indirect relationship between cyberloafing and the sense of meaning of life. Meanwhile, demographic variables (gender, grade, experience of using the network) were controlled when we examined the mediating effect and moderating effect. The bootstrap confidence intervals (CIs) determine whether the effects in Model 4 and Model 7 are significant based on 5,000 random samples. An effect is regarded as significant if CIs do not include zero.

## Results

### Common method bias

The Harman single-factor test was used to test common method deviation (37). The results revealed that the mutation rate interpretation of the first factor was 28.63%, which was less than the critical value of 40%, indicating that there was no obvious deviation of common method in this study.

We compared our hypothesized model (i.e., model 4, the baseline four-factor model) with a three-factor model (i.e., model 3, combining cyberloafing and state anxiety), a two-factor model (i.e., model 2 combining cyberloafing and state anxiety and combining the sense of meaning of life and psychological flexibility), and a one-factor model combining all items (i.e., model 1) (Table 1). Considering the changes in chi-square (i.e., χ^2^), two major fit indicators [i.e., comparative fit index (*CFI*) and incremental fit index (*IFI)*], and root mean square error of approximation (*RMSEA*), our hypothesized four- factor model [with χ^2^*/df* = 2.41, *IFI* = 0.95, *CFI* = 0.95, and *RMSEA* = 0.04] showed better fit than other alternative models (44, 45). *RMSEA* < 0.05, and χ^2^*/df* < 3 indicated good model fit (46), while the other indices such as *CFI, IFI* > 0.90 can be construed as an acceptable fit (47). Therefore, the discriminant validity of the constructs was confirmed. This suggests that the participants of our survey could distinguish the focal constructs clearly.

**Table 1:** Results of confirmatory factor analysis of the measurement models.

**Measurement models**	***χ** ^**2**^*	***df***	***χ** ^**2**^ **/df***	***RMSEA***	***IFI***	***CFI***
Model 1: One-factor (combined all items into one factor)	14,999.23	1,080	13.89	0.12	0.55	0.55
Model 2: Two-factor (combined CL and SA into one factor, and combined ML and PF into one factor)	11,389.34	1,079	10.56	0.10	0.67	0.67
Model 3: Tdree-factor (combined CL and SA into one factor)	9,096.35	1,077	8.45	0.09	0.74	0.74
Model 4: Four-factor	2,592.34	1,074	2.41	0.04	0.95	0.95

*CL, Cyberloafing; SA, State anxiety; ML, Meaning of life; PF, Psychological flexibility*.

### Descriptive statistics and correlation analysis

Mean value, standard deviations, Cronbach's alpha, and correlation coefficient of the variables are shown in Table 2. Correlation analysis showed that cyberloafing is significantly positively correlated with state anxiety (*r* = 0.33, *p* < 0.01) and negatively correlated with the sense of meaning of life (*r* = −0.16, *p* < 0.01); state anxiety is significantly negatively correlated with the sense of meaning of life (*r* = −0.20, *p* < 0.01). Thus, these results preliminarily support the subsequent regression analysis.

**Table 2:** Descriptive statistics and correlation analysis.

**Variable**	***M***	***SD***	**1**	**2**	**3**	**4**	**5**	**6**
1 Gender	1.71	0.45						
2 Age	18.67	1.36	−0.03					
3 Internet	2.23	0.65	−0.13**	0.11**				
4 Cyberloafing	2.56	1.06	0.00	0.00	0.07*			
5 State anxiety	2.56	0.48	0.05	−0.01	−0.04	0.33**		
6 Psychological flexibility	3.31	0.75	0.00	−0.01	0.09**	−0.20**	−0.55**	
7 Meaning of life	3.57	0.46	−0.07*	0.02	0.13**	−0.16**	−0.20**	0.19**

*p <0.05; **p <0.01.

### Hypotheses testing

Model 4 (a simple mediation model) in the SPSS expansion macro-PROCESS prepared by Hayes (48) was used to test the mediating effect of state anxiety on the relationship between cyberloafing and the sense of meaning of life. Cyberloafing was a significant predictor of the state anxiety (β = 0.34, SE = 0.01, *p* < 0.01) and the sense of meaning of life (β = −0.17, SE= 0.03, *p* < 0.01). State anxiety was a significant predictor of the sense of meaning of life (β = −0.20, SE = 0.03, *p* < 0.01). Results of the bootstrapping test (β = −0.16, SE = 0.03, *p* < 0.01) supported that CI did not contain zero. Therefore, the hypothesis that state anxiety plays a partial mediating role in the relationship between cyberloafing and the sense of meaning of life was supported. The direct (−0.11) and mediated (−0.05) prediction effects accounted for 68.75 and 31.25% of the overall effect, respectively.

**Figure 2:**
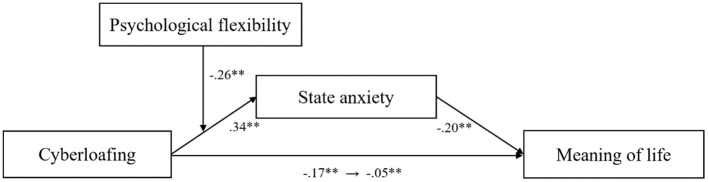
The moderated mediation model.

In the second step, we employed Model 7 in the SPSS extension macro, and the moderated mediation model was tested. As shown in Table 3, after inputting psychological flexibility into the model, the interaction between cyberloafing and psychological flexibility was a significant predictor of state anxiety (Cyberloafing × Psychological flexibility: β = −0.26, SE = 0.03, *p* < 0.01), indicating that psychological flexibility moderated the relationship between cyberloafing and state anxiety (Model 1). All results are presented in Figure 2.

**Table 3:** Moderated mediation effect analysis.

	**Model1 (criterion: state anxiety)**				**Model2 (criterion: meaning of life)**			
	***β***	**SE**	***p-*** **value**	**95% CI**	***β***	**SE**	***p-*** **value**	**95% CI**
Control variables								
Gender	0.13*	0.05	0.02	[0.02, 0.24]	−0.10	0.07	0.15	[−0.24, 0.03]
Age	−0.01	0.02	0.69	[-0.04, 0.03]	0.00	0.02	0.87	[−0.04, 0.05]
Internet	0.00	0.04	0.91	[-0.07, 0.08]	0.19**	0.05	<0.01	[0.09, 0.28]
Independent variable								
Cyberloafing	0.26**	0.03	<0.01	[0.21, 0.31]	−0.11**	0.03	<0.01	[−0.18,−0.05]
Mediator								
State anxiety					−0.16**	0.03	<0.01	[−0.22,−0.09]
Moderator								
Psychological flexibility	−0.37**	0.03	<0.01	[−42,−0.31]				
Interaction term								
Cyberloafing × Psychological flexibility	−0.26*	0.03	<0.01	[−0.31,−0.21]				
*R^2^*	0.42	0.07
*F*	114.12**	14.01**

*p <0.05; **p <0.01.

**Figure 3:**
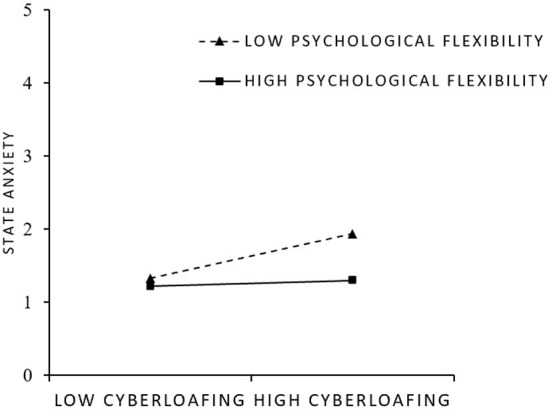
Schematic diagram of interaction effect (state anxiety).

In addition, we plotted the interaction effects at different levels (i.e., +1 SD or −1 SD) of psychological flexibility using the recommendation of Aiken and West (49). Figure 3 shows that cyberloafing is more positively related to state anxiety when psychological flexibility is low rather than high. Accordingly, the hypothesis that the moderating effect of psychological flexibility on the cyberloafing–state anxiety relationship was supported.

We further estimated the conditional indirect effect of cyberloafing on the sense of meaning of life *via* state anxiety across levels of psychological flexibility by bootstrapping the bias-corrected CI. The results are presented in Table 4. The indirect effect of cyberloafing on the sense of meaning of life through state anxiety was stronger and significant at a low level of psychological flexibility (effect size = −0.08, 95% bias-corrected CI from−0.13 to−0.03), but was weaker at a high level of psychological flexibility (effect size = −0.00, 95% bias-corrected CI from−0.01 to 0.01). Thus, hypothesis that psychological flexibility moderates the mediating effect of state anxiety between cyberloafing and sense of life meaning was supported.

**Table 4:** Results for conditional indirect effect across levels of psychological flexibility.

**Level**	**Effect size**	**Boot SE**	**LL 95% CI**	**UL 95% CI**
*M - SD*	−0.08	0.02	−0.13	−0.03
*M*	−0.04	0.01	−0.07	−0.02
*M + SD*	< -0.01	0.01	−0.02	0.01

## Discussion

### Result analysis

Based on the social displacement hypothesis and the stress transactional model, this paper discusses the impact of college students' cyberloafing on the sense of meaning of life through state anxiety.

Firstly, this study found that cyberloafing has a significant negative effect on college students' sense of meaning of life. With the application of the Internet in the classroom, it brings convenience to learning, but it also brings some disadvantages. Cyberloafing on the Internet brings temporary pleasure, but it causes deeper academic problems (50). This is due to the fact that cyberloafing while studying has occupied a portion of college students' time which should have been devoted in academic and social activities; the consumption of scarce attention resources by cyberloafing has resulted in a sense of loss and helplessness (16), which creates a negative impact on the sense of meaning of life (27, 28). This finding has verified previous research (7) to a certain extent. More importantly, problematic network behaviors could cause college students to lose the pursuit of life and the meaning of life (18), which may be one of the reasons why contemporary youth lack a sense of meaning in life. Therefore, how to guide college students to use the Internet more reasonably in class, and better allocate attention and study time, is a problem that educators have to face.

Secondly, our results show that state anxiety partially mediates the relationship between cyberloafing and the sense of meaning of life. That is, as a problematic network using behavior (3), cyberloafing consumes resources and tends to cause college students' negative emotions such as stress and state anxiety, thus reducing their sense of meaning of life. This conclusion is consistent with the findings of Becker et al. (13), that is, cyberloafing could lead to higher level of social anxiety among college students. With the occurrence of social anxiety, college students' subjective wellbeing and sense of meaning of life will also decline (24). Therefore, it is of great significance for college students to maintain a stable and peaceful mental state in their daily study and life and learn to reduce their anxiety.

Finally, psychological flexibility can moderate the relationship between cyberloafing and state anxiety. In particular, by testing the moderated mediation model, this study further shows that psychological flexibility negatively moderates the mediating effect of state anxiety on the relationship between cyberloafing and sense of meaning of life. Specifically, individuals with high level of psychological flexibility have more effective adaptive and coping strategies and can better recover from the stressful situation of cyberloafing (36), therefore, their level of state anxiety can be reduced and their sense of value and meaning in life can be maintained and improved.

### Theoretical significance

The theoretical significance of this paper can be reflected in the following three aspects. First, extant researches on cyberloafing at home and abroad mainly focus on the field of organizational behaviors within firms, while there are few empirical studies carried out in the context of the educational situation. This study focuses on the impact of college students' cyberloafing on the sense of meaning of life, which has expanded the research scope on cyberloafing and has revealed a hypothesized internal mechanism of this negative impact; this has, to some extent, contributed to the development of the cyberloafing field. Second, although there are numerous studies on the sense of meaning of life both at home and abroad, previous studies mainly focus on the impact of the sense of meaning of life on other variables, while hardly has any studies turn their research lenses toward the antecedent variables of the sense of meaning of life and the individual factors affecting the generation of the sense of meaning. This study reveals a possible mechanism of cyberloafing on the sense of meaning of life based on the social displacement theory, which has provided a brand-new perspective for the study of the influencing factors of meaning of life. Third, this study has introduced the variable of psychological flexibility to explore the causes of the sense of meaning of life, which has not only enriched the existing research on the sense of meaning of life, but also has provided a reasonable approach to construct the theoretical system of the sense of meaning of life.

### Practical implication

Firstly, this study found that college students' cyberloafing will negatively affect their sense of meaning of life. It is extremely necessary to guide them to use the Internet reasonably and moderately, enhance their self-control ability, and remind them of the potential harm of excessive cyberloafing; meanwhile, it is also necessary to exert the influence of mutual support and encouragement of peers to contribute to the formation of a good learning atmosphere and reduce cyberloafing behavior.

Secondly, the important intermediary mechanism of state anxiety found in this study requires educators to pay active attention to the mental health and emotional state of college students, and effectively intervene their negative emotions through activities such as individual or group psychological counseling, emotional management lectures, mental health therapies and so on, so as to enhance college students' ability of emotional adjustment and provide supportive measures for the maintenance of their good emotional state.

Finally, this study also shows that there exist individual disparities as regard to the mechanism of cyberloafing. Individuals with low level of psychological flexibility tend to adopt more rigid and negative coping styles, resulting in more anxiety; while individuals with high level of psychological flexibility tend to more easily give up their defense against the current situation, feel and experience their current thoughts and feelings, and alleviate their own anxiety. College students are advised to avoid falling into the trap thinking and complaining about the negative results brought by cyberloafing; instead, they should accept the current situation, switch to a more positive way of thinking, and maintain and enhance their own sense of meaning of life. Therefore, educators are advised to provide relevant trainings aiming at enhancing the psychological strength for college students with low level of psychological flexibility, and carry out necessary activities of psychological adjustment, through which the psychological flexibility of such college students can be improved.

### Limitations and prospects

First, the utilization of time-lagged data in this paper cannot guarantee very clear evaluation of the causal relationship among variables, which would inevitably result in inaccurate research conclusions. Future research is suggested to adopt a longitudinal research method to reduce homologous deviation and further improve the validity of conclusions. Second, due to the limitations in time, manpower and resources, the scope of this study is relatively limited. Future research can improve the external validity of conclusions by expanding the source and scope of samples. Third, this study found that state anxiety plays a partial mediating role between cyberloafing and sense of life meaning, which indicates the existence of other mediating variables. Future research can be carried out to explore other types of variables, such as satisfaction from basic psychological needs and so on.

## Data availability statement

The raw data supporting the conclusions of this article will be made available by the authors, without undue reservation.

## Ethics statement

The studies involving human participants were reviewed and approved by the Ethics Committee of Communication University of Zhejiang. The patients/participants provided their written informed consent to participate in this study.

## Author contributions

QL was in charge of the formulation of the general research topic and the proposing of the theoretical hypothesis. BX analyzed the data and conceptualized the models. HZ and WW collected the data. XW supervised the project. All authors contributed to the article and approved the final manuscript.

## Funding

This research was supported by the General Project of National Social Science Foundation in China in 2020 [Grant No. 20BSH130], the Youth Project of Humanities and Social Sciences of the Ministry of Education in China in 2017 [Grant No. 17YJC190012], and the Zhejiang Federation of Humanities and Social Sciences Research Project in 2017 [Grant No. 2017Z10].

## Conflict of Interest

The authors declare that the research was conducted in the absence of any commercial or financial relationships that could be construed as a potential conflict of interest.

## Publisher's Note

All claims expressed in this article are solely those of the authors and do not necessarily represent those of their affiliated organizations, or those of the publisher, the editors and the reviewers. Any product that may be evaluated in this article, or claim that may be made by its manufacturer, is not guaranteed or endorsed by the publisher.
